# Insurance Notes

**Published:** 1923-04

**Authors:** 


### NATIONAL HEALTH INSURANCE NOTES.

### HOW SHALL THE DOCTORS BE PAID?

SOME people are looking for salvation from the ills of the panel system in a change of method of paying the doctor. If, they suggest, the doctor is paid for each service rendered, instead of getting a fee for each person on his list, whether that person is well or ill, we shall be approximating more closely to the conditions of private practice. This, no doubt, sounds very attractive. But its advocates have to make up their minds whether they are going to pay for an unlimited number of services, stated by the several doctors to be necessary, or whether this number is to be carefully supervised, and if so, how? And who is going to do all the book-keeping? One fundamental advantage which the capitation system of payment has over other systems is that it is to the doctor's advantage to get his patient well and to keep him well.

### CHANGING DOCTORS.

One of the advocates of payment of doctors for each service rendered instead of for each person on his list is Mr. J. P. Lewis, the Secretary of the Hearts of Oak Society. In a long letter to the *Times* recently he adduced a series of arguments all of which would be found, on examination, to ascribe to the attendance system of payment the particular merit that an insured person could change his doctor at any time. As a matter of fact, this has no necessary connection at all with that method of payment. It is just as feasible under the capitation system of payment. What Mr. Lewis and others who think with him really want to secure is just this right to change doctors at any time, and it is one which it is hardly worth the while of the doctors' representatives to resist if it is pressed. In practice, it would not cause much disturbance, and they need not be alarmed by the illustration with which Mr. Lewis rather prejudices his own case—that of the insured person who changes his doctor six times in six weeks.

### FACILE CERTIFICATION.

If the Societies press for unrestricted change of doctor at any time, they will have to reckon with the risk which the system entails of a patient giving up a doctor who is strong enough to refuse certificates of incapacity and going to another who gives them with greater facility. It is a risk which will involve an additional number of references to referees, and might necessitate some arrangement whereby the doctor who is left may be asked to certify whether, in his opinion, the patient was incapable of work at the time of leaving. There may be an incidental advantage in the accumulation of evidence leading to the ultimate elimination of the too-popular doctor.

### THE SKETCHY PARAGRAPH.

In the *Sunday Pictorial*, under the heading "Panel System Doomed," we read:—"I understand there is little doubt that at the end of this year the panel system under the Health Insurance Act will be entirely discontinued, and a system of payment by visit substituted. The free choice of doctors will be restored, and incidentally the Exchequer will save £7,000,000 a year." This is the sort of sketchy paragraph which always stops shorts at the interesting point. If the writer would give some indication of how this wonderful saving is to be made by paying doctors for each visit, he would be doing a real public service. And, incidentally, he would be performing a conjuring trick, as the present cost to the Exchequer is under two millions a year.

### BLACK SHEEP.

Humanly speaking, whatever farmers may say, declares Professor Gray, in a recently-published pam phlet,* delightfully written, there are black sheep in every flock, and there have been societies in which members needed protection against their own committee of management. This is one reason, and there are others, why it is impossible for the State to restrict its activities in connection with the Insurance scheme to the enforcement of the payment of contributions. It is a change to read for once in a way of the existence of black sheep somewhere else than in the medical profession.

### SELF-GOVERNMENT.

Professor Gray is very pleasantly outspoken on this subject, which, of course, he knows thoroughly. One Government Department, he says, may lead the insured person to the refreshing waters of self-determination, but twenty departments cannot make him drink. It is no secret that, although in the matter of quorums the rules of societies have been reduced to the verge of absurdity, numbers of these societies have the utmost difficulty in getting a quorum where it is not possible to mobilise a solid phalanx of typistsfor the purpose.

### SIMPLIFICATION NEEDED.

Owing to the well-meant efforts of legislators in 1911, in which every section, and every sub-section, of the House of Commons took a hand, the Insurance Act, when it became law, was, says Mr. Gray, of a complexity positively dementing. He urges the need for codification with all the amending Acts, accompanied by simplification, even if it involves some sacrifice in logic and abstract justice, and a pruning of all clauses which, in sentiment admirable, have been found to be of no practical significance.

### DOCTORS IN SCOTLAND.

In Scotland, at any rate, it is difficult to speak of panel doctors as of a class distinct from the medical profession as a whole. It appears from a return recently published by the British Medical Association that 89·5 per cent. of the general practitioners in Scotland are on the panel.

* "Some Aspects of National Health Assurance," by Alexander Gray, Professor of Political Economy, University of Aberdeen. P. S. King & Son, Westminster. 2s.

